# Tetramethylpyrazine Preserves the Integrity of Blood-Brain Barrier Associated With Upregulation of MCPIP1 in a Murine Model of Focal Ischemic Stroke

**DOI:** 10.3389/fphar.2021.710358

**Published:** 2021-07-28

**Authors:** Zhuqing Jin, Jian Liang, Pappachan E. Kolattukudy

**Affiliations:** ^1^ School of Basic Medical Sciences, Zhejiang Chinese Medical University, Hangzhou, China; ^2^ Burnett School of Biomedical Sciences, University of Central Florida College of Medicine, Orlando, FL, United States

**Keywords:** tetramethylpyrazine, MCPIP1, middle cerebral artery occlusion, reperfusion, blood-brain barrier

## Abstract

Tetramethylpyrazine (TMP), a prominent ingredient of Chinese herb Ligusticum chuanxiong Hort, is known to suppress neuroinflammation and protect blood-brain barrier (BBB) integrity. We investigated whether monocyte chemotactic protein-induced protein 1 (MCPIP1, also known as Regnase-1), a newly identified zinc-finger protein, plays a role in TMP-mediated anti-inflammation and neuroprotection. Male C57BL/6 mice were subjected to focal cerebral ischemia induced by middle cerebral artery occlusion (MCAO) for 2 h, followed by reperfusion for 24 h. TMP (25 mg/kg or 50 mg/kg) or vehicle was administered intraperitoneally 12 h before and post MCAO. The TMP significantly upregulated MCPIP1 in the ischemic brain tissues and effectively inhibited extravasation of fluorescein isothiocyanate (FITC)-dextran, resulting in attenuation of brain edema. These effects of the TMP were associated with a significant reduction in levels of inflammatory cytokines tumor necrosis factor (TNF)-α, interleukin (IL)-1β, IL-6, and MMP-9 in the ischemic brain tissues. The TMP upregulated the expression of MCPIP1 in primary cultures of neurons and protected against oxygen–glucose deprivation-induced neuron death, while this neuroprotective effect of TMP was abolished by knockdown of MCPIP1 using MCPIP1-specific siRNA. These results suggest that preservation of BBB integrity by TMP is associated with its anti-inflammatory activity. The effect of TMP is mediated, at least in part, *via* upregulation of MCPIP1 in the ischemic brain.

## Introduction

Traditional Chinese herb *Ligusticum chuanxiong Hort* is widely used for the treatment of stroke and cardiovascular diseases due to its proven clinical benefits. (Ran et al., 2011). To date, many bioactive components of this herb have been identified, of which tetramethylpyrazine (TMP) is recognized as a prominent bioactive ingredient and has been widely used as a possible intervention against ischemic brain injury due to its anti-inflammatory, antioxidant, and anti-apoptosis effects. (Sun et al., 2012; Kao et al., 2013; Yang et al., 2017). TMP was reported to reduce the infarct volume, neurological score and brain edema in animal models of ischemic cerebral injury, (Xiao et al., 2010; Kao et al., 2013), which has been further confirmed by multiple *in vitro* studies. (Li et al., 2010; Zhao et al., 2016). Recently, TMP has been shown to improve inflammation-mediated permeability blood-brain barrier (BBB), a key event that leads to brain edema and the progression of neurological dysfunction, in animal models of traumatic brain injury (Wang et al., 2017) and focal cerebral ischemia and reperfusion (Tan et al., 2015). However, the underlying mechanisms of TMP on the improvement of permeability of the BBB, have not been fully elucidated.

Monocyte chemoattractant protein (MCP)-1 is a pro-inflammatory chemokine, whose expression is found in the ischemic brain in mice (Dimitrijevic et al., 2006; Yao and Tsirka, 2014). An increase of pro-inflammatory cytokines, such as tumor necrosis factor (TNF)-α, interleukin (IL)-6, MCP-1 and IL-1β, has been reported to promote the permeability of the BBB. (Strecker et al., 2011). In addition, matrix metalloproteinases (MMPs), such as MMP-9 induced by the pro-inflammatory cytokines (i.e., TNF-α, IL-1β), can degrade the extracellular matrix and tight junction proteins, resulting in the disruption of the BBB (Yang and Rosenberg, 2011).

Therefore, attenuation of the neuroinflammation induced by ischemic stroke would protect the integrity of the BBB, prevent the progression of ischemic brain injury. Thus, identification of key molecules associated with the anti-inflammatory activity of TMP is a promising strategy to develop novel therapeutics against the BBB breakdown occurring in ischemic stroke.

We recently showed that monocyte chemotactic protein-induced protein 1 (MCPIP1; also known as Regnase-1), originally discovered as a zinc-finger protein in human peripheral blood monocytes treated with MCP-1 (Zhou et al., 2006), is a suppressor of inflammation (Matsushita et al., 2009; Liang et al., 2010). MCPIP1 is also expressed in endothelial cells and microglia upon lipopolysaccharide (LPS) stimulation, (Liang et al., 2008; Niu et al., 2008), and participates in LPS preconditioning and electroacupuncture-induced ischemic brain tolerance (Liang et al., 2011; Jin et al., 2013). Importantly, MCPIP1-deficient mice display an increased permeability of the BBB following transient focal ischemia (Jin et al., 2019a). Therefore, we speculated that MCPIP1 could mediate the anti-inflammatory activity of TMP, thus maintaining the integrity of the BBB during ischemic stroke. To test this hypothesis, the induction of MCPIP1 by TMP was measured and the relevant mechanisms associated with the preservation of the integrity of BBB by TMP was investigated in a murine model of focal ischemic stroke and in primary cultures of cortical neurons *in vitro*. Here, we show that TMP attenuates the permeability of the BBB is, at least in part, *via* upregulation of the MCPIP1 in the ischemic brain.

## Materials and Methods

### Animals and Focal Ischemic Model

Male C57BL/6 mice (6–8 weeks old, body weight 22–25 g) were obtained from the transgenic animal facility on the main campus of University of Central Florida. All experimental procedures were approved by the Institutional Animal Care and Use Committee of University of Central Florida (Approval Number 14–29). The study was conducted in accordance with the Basic and Clinical Pharmacology and Toxicology policy for experimental and clinical studies (Tveden-Nyborg et al., 2018). Mice were initially anesthetized with 3% of isoflurane and maintained with 1.2% of isoflurane mixed with oxygen by the facemask. A transient middle cerebral artery occlusion (MCAO) was produced in mice by filament occlusion of the right middle cerebral artery (MCA) as we described previously (Jin et al., 2013). Rectal temperature was maintained at 37 ± 0.5°C during the procedure with a heating pad. MCA was occluded for 2 h followed by reperfusion for 24 h. The foot fault evaluation was performed 2 h post MCAO to ensure neurological deficits induced by MCAO.

TMP 25 mg/kg or 50 mg/kg or vehicle was administered intraperitoneally 12 h before and 2 h post MCAO (n = 10–12 per group, total n = 60). The TMP was purchased from Sigma (St. Louis, MO, United States) and dissolved in ethanol-water solution. The dose of TMP was chosen based on the previous study (Chen et al., 2017).

### Assessment of the BBB Disruption

To assess the protective effects of TMP on the BBB integrity, a previously described fluorescein isothiocyanate (FITC)-dextran assay was performed (Natarajan et al., 2017). FITC-dextran (500 mg/kg, Sigma, United States) was administered intravenously *via* tail vein to the mice at the end of reperfusion. The mice brains were then removed and fixed in 4% paraformaldehyde at 4°C for 24 h. The brains were coronally sectioned and scanned under a fluorescence microscope (Leica TCS SP5) for the assessment of FITC-dextran extravasation. Three fields per section and five sections were analyzed per animal.

### Brain Water Content Measurement

At the end of reperfusion, the mice were sacrificed by cervical dislocation and the mice brains were rapidly removed. The brains were weighed to obtain the wet weight and then dried at 105°C for 24 h and reweighed. Percent brain water content was calculated as 100× (wet weight−dry weight)/wet weight.

### Assessment of Inflammatory Cytokines by Quantitative Real-Time PCR

mRNA levels for MCPIP1, TNF-α, IL-6, MCP-1, and IL-1β were measured by quantitative real time PCR as we described in our previous publication (Jin et al., 2013). mRNA levels for these genes were quantified by the delta cycle time method and normalized to the β-actin.

### Immunoblots

The protein expression levels of MCPIP1 and MMP-9 in the brain tissues of ischemic penumbra were detected by immunoblot analysis as previously described (Jin et al., 2013), as ischemic penumbra is considered salvageable if re-perfused. Proteins extracted from the ischemic penumbra were separated by the SDS-PAGE and transferred onto the nitrocellulose membranes in transfer buffer containing 0.1% SDS. After blocking, the membranes were incubated with the primary antibodies against MMP-9 (rabbit, #3852, Cell Signaling, United States) or MCPIP1 (rabbit, ab97910, abcam, United States) at a 1:1,000 dilution at 4°C overnight with gentle shaking. The membranes were incubated with an appropriate secondary antibody (1:2,000 dilution, goat anti-rabbit IgG-HRP, sc-2030, Santa Cruz Biotechnology, United States) for 1 h, and then visualized with SuperSignal West Pico Chemiluminescent Substrate (Pierce, United States). The intensity of immunoreactive bands was quantified by AlphaImage 2,200 (AlphaInnotech, United States) and normalized to the loading control β-actin. The protein expression levels were calculated as fold changes over the sham-treated group.

### Oxygen-Glucose Deprivation in Primary Cultures of Mouse Cortical Neurons

Primary culture of mouse cortical neurons was prepared as previously reported (Hilgenberg and Smith, 2007) with some modifications. Briefly, the mouse brain cortex was digested in 0.25% trypsin at 37°C for 10 min. The cell suspension was centrifuged at 1,000 rpm for 5 min. The pellet was resuspended in Neurobasal-A/B-27 medium (Thermo Fisher Scientific) with 0.25 mmol/L Glutamax-1, 40 U/mL penicillin, 40 μg/ml streptomycin, and 10% dialyzed horse serum. Cells were plated in poly-L-lysine-coated 24-well plates at 6 × 10^5^ cells per well, and cultured at 37°C in a humidified chamber containing 5% CO_2_.

Transfections of MCPIP1 siRNA (4,390,771, Life Technologies) or control siRNA (4,390,843, Life Technologies) were performed as previously reported (Liang et al., 2008) using Lipofectamine 2000 reagent (Invitrogen) according to the manufacturer’s instructions. 24 h later after 25 nM of the siRNA transfection, the TMP (10 μM) was added to the culture medium for 12 h, and the cells were subjected to OGD protocol as previously described (Gong et al., 2014). Briefly, the cells were cultured with glucose-free medium with 95% N_2_, 5% CO_2_ at 37°C for 120 min. The culture medium was then replaced with normal culture medium and incubated under normal conditions for 24 h. Cells without OGD were used as controls.

### MTT Assay

At the end of OGD, 10µl of MTT (Sigma-Aldrich, United States) was added (final concentration, 0.5 mg/ml) to the cultured cells for 3 h. Cells were then lysed with 100 μL of 10% SDS in 0.01 N HCl for 16 h. Absorbance values were read in an automatic microplate reader per the manufacturer’s instructions.

### Statistical Analysis

Data are presented as mean ± standard deviation (SD). Statistical comparisons were performed using an unpaired two-tailed Student’s -test for two groups and one-way analysis of variance (ANOVA) followed by the Tukey or Dunnett test for more than two groups. Statistical analysis was performed using the SPSS V.26.0 software (SPSS; Chicago, IL, United States). A *p* value of <0.05 was considered statistically significant.

## Results

### TMP Upregulates MCPIP1 Expression in the Murine Brain

**FIGURE 1:**
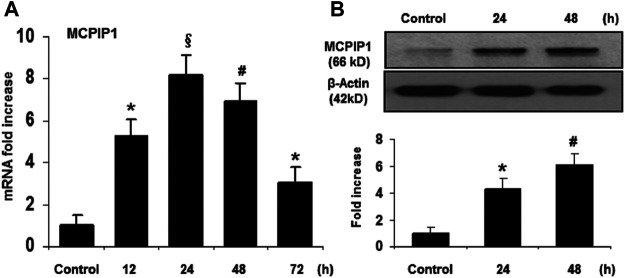
TMP upregulates MCPIP1 expression in the murine brain. The C57BL/6 mice were treated with TMP 25 mg/kg or vehicle administered intraperitoneally, and expression of MCPIP1 was measured at 12, 24, 48, and 72 h after TMP administration. **(A)** mRNA levels for MCPIP1 in the mouse brain cortex were measured by qRT-PCR, and expressed as fold changes of untreated controls. *p < 0.05, ^#^ *p* < 0.01, ^§^ *p* < 0.001 versus untreated controls; n = 5 per timepoints. **(B)** Protein levels for MCPIP1 in mouse brain were measured by immunoblots **(*Upper panel*)**, and the immunoreactivity bands were quantified by densitometry analysis normalized to *β*-actin, and expressed as fold changes over the untreated controls **(*Lower panel*)**. *p < 0.05, ^#^ *p* < 0.01, ^§^ *p* < 0.001 versus untreated control. n = 5 per timepoints.

To determine whether MCPIP1 contributes to anti-inflammatory effects of TMP in a murine model of ischemic stroke, we examined the mRNA levels of MCPIP1 in the mice brains upon TMP treatment. As shown in Figure 1A, mRNA levels for MCPIP1 were detected in the areas of the brain cortex at 12 h and reached 8-fold at 24 h and began to decline by 48 h after the TMP treatment. Consistently, protein levels of MCPIP1 in the brain tissues were significantly elevated by the TMP treatment, 4.3-fold increase at 24 h and remained the higher levels at 48 h compared to the vehicle-treated controls (Figure 1B).

### TMP Improves BBB Permeability and Brain Edema in Ischemic Brain Injury

**FIGURE 2:**
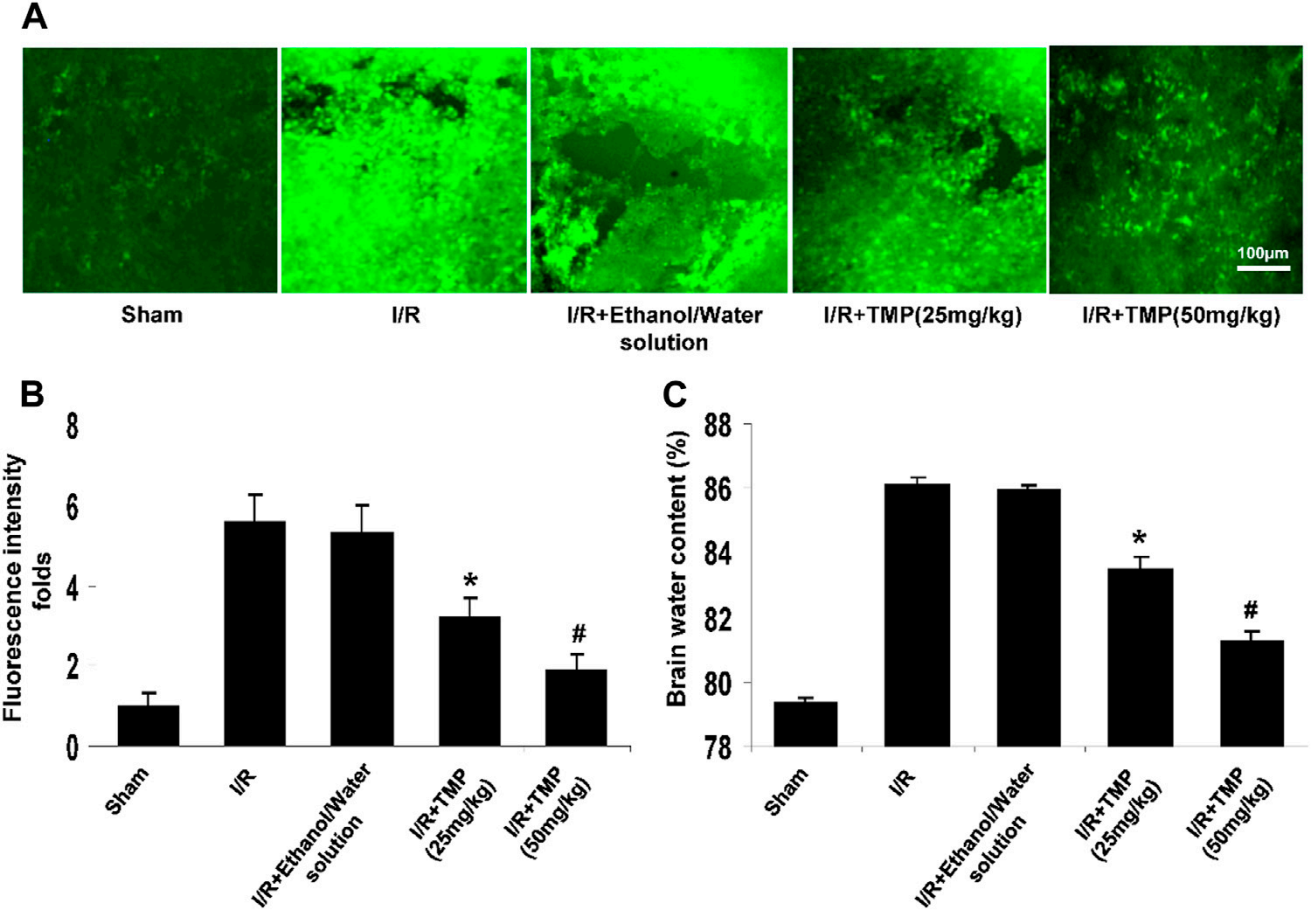
TMP improves BBB permeability and brain edema in ischemic brain injury. The C57/BL mice were treated with TMP 25 mg/kg or 50 mg/kg or vehicle administered intraperitoneally 12 h before and after 2 h of MCAO, followed by 24 h reperfusion. **(A)** Representative photomicrographs of brain sections showing extravasation of the FITC-dextran identified by fluorescence staining (*green*) in peri-infarct cortical areas. **(B)** The fluorescence intensity of FITC-dextran was quantified by a fluorescence microscope (Leica TCS SP5). *p < 0.05, ^#^ *p* < 0.01 versus the sham-operated controls. **(C)** TMP attenuated cerebral ischemia/reperfusion-induced brain edema in a dose-dependent manner. *p < 0.05, ^#^ *p* < 0.01 versus the I/R group. n = 6 per group.

The disruption of BBB contributes to cerebral edema and the progression of infarction volume and neurological deficits (Rosenberg, 2012). Since TMP was shown to reduce infarct volume in animal models of stroke (Xiao et al., 2010; Kao et al., 2013), we examined if the TMP attenuates the BBB permeability caused by cerebral ischemia/reperfusion. The BBB permeability was assessed using FITC-dextran by histochemical analysis and the results showed that the focal ischemia/reperfusion induced FITC-dextran extravasating into the peri-infarct cortex of the brain compared to that seen in the sham-operated controls, whereas the extravasation of FITC-dextran was significantly reduced by the TMP treatment in a dose-dependent manner (Figure 2A). Measurement of fluorescence intensity of the extravasated FITC-dextran showed that the leakage of FITC-dextran was markedly increased up to 6.0-fold compared to that seen in the sham-operated controls, and this increase in fluorescence intensity of FITC was reduced by the TMP treatment in a dose-dependent manner (Figure. 2B). Consistently, the brain edema was significantly attenuated in the TMP-treated mice compared with the vehicle-treated mice (Figure. 2C).

### TMP Inhibits Proinflammatory Gene Expression in the Ischemic Brain

**FIGURE 3:**
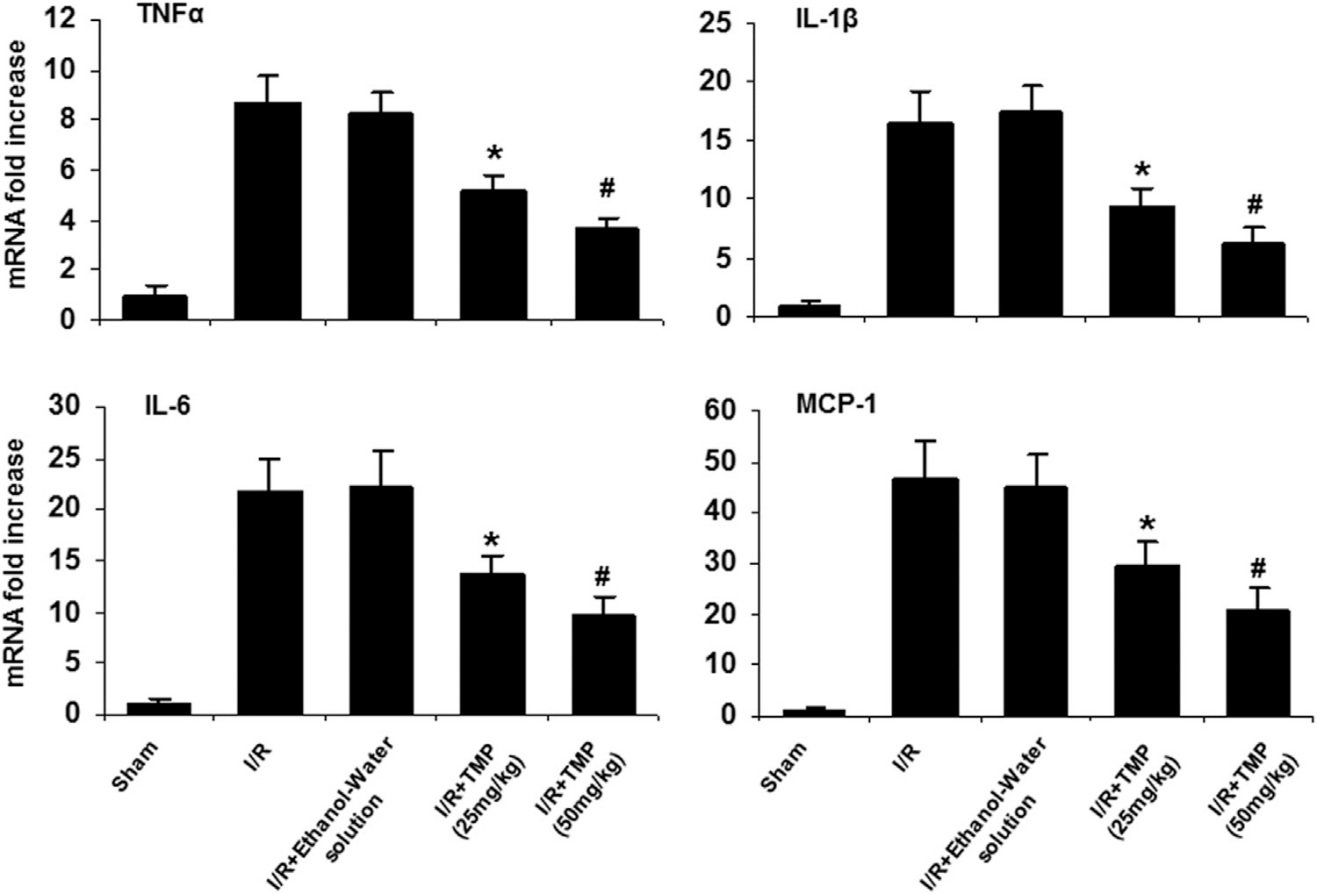
TMP reduces expression of pro-inflammatory cytokines in the ischemic murine brain. The C57/BL mice were treated with TMP 25 mg/kg or 50 mg/kg or vehicle administered intraperitoneally 12 h before and after 2 h of MCAO, followed by 24 h reperfusion. mRNA levels of TNFα, IL-1β, IL-6, and MCP-1 were measured by qRT-PCR and expressed as fold changes of sham-operated controls. Elevated mRNA levels of TNFα, IL-1β, IL-6, and MCP-1 were observed in the mice brains subjected to cerebral ischemia/reperfusion injury compared to the sham-operated mice. The elevated mRNA levels of these cytokines in the ischemic brain were significantly reduced by treatment with TMP in a dose-dependent manner. *p < 0.05, ^#^ *p* < 0.01, n = 6 per group.

Proinflammatory cytokines can lead to the recruitment and activation of inflammatory cells (neutrophils/macrophages), resulting in the degradation of the extracellular matrix and subsequent disruption of the BBB (Alluri et al., 2015; Esenwa and Elkind, 2016). Given that TMP has anti-inflammatory properties and could effectively improve BBB permeability, we examined the possible effects of TMP on the expression of pro-inflammatory cytokines. mRNA levels for TNF-α, IL-1β, IL-6, and MCP-1 in the ischemic brain were assessed by qRT-PCR. As shown in Figure 3, mRNA levels of TNF-α, IL-1β, IL-6, and MCP-1 were significantly elevated in the ischemic brain tissues compared with the sham-operated mice, and the expression of these cytokines was markedly reduced in the TMP-treated group compared with the vehicle-treated group.

**FIGURE 4:**
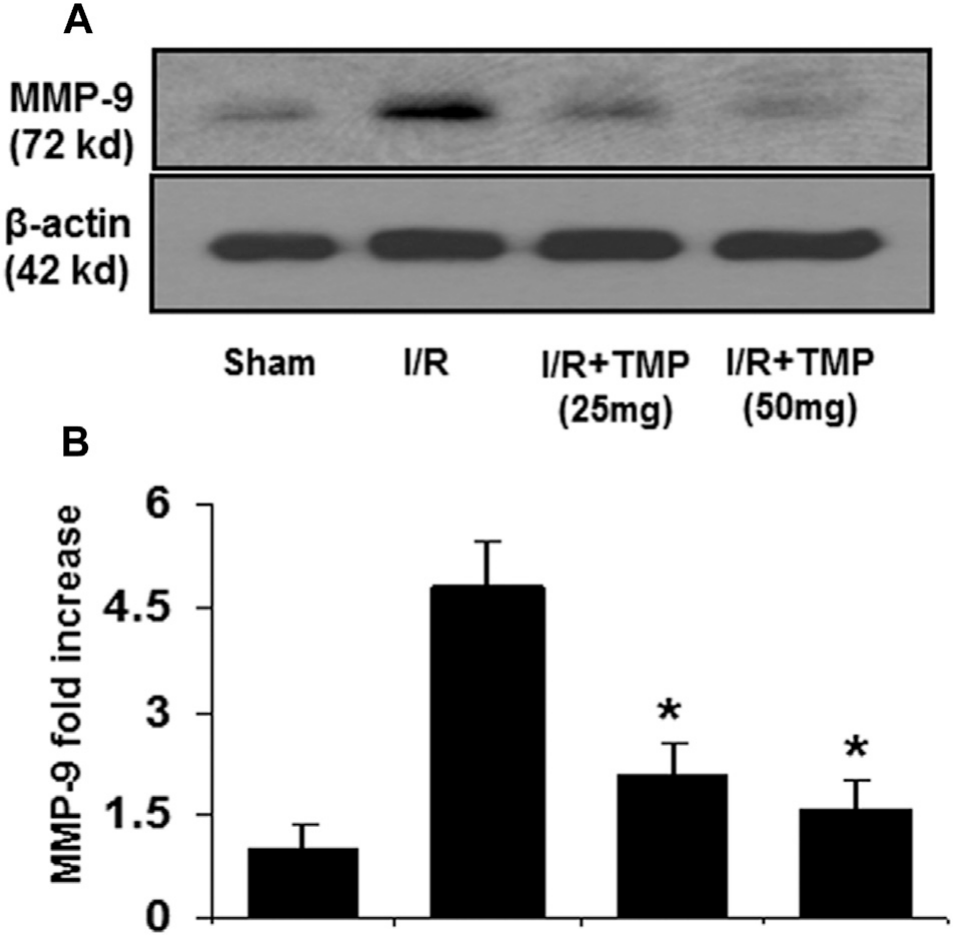
TMP inhibits the expression of MMP-9 in the ischemic murine brain. The C57/BL mice were treated with TMP 25 mg/kg or 50 mg/kg or vehicle administered intraperitoneally 12 h before and after 2 h of MCAO, followed by 24 h reperfusion. Proteins extracted from the ischemic brains were subjected to immunoblot analysis with an antibody against MMP-9. **(A)** Representative photomicrographs show MMP-9 immunoreactivity in the ischemic brains in the different treatment groups. **(B)** The bar graphs represented the quantification of MMP-9 expression by densitometric analysis of the immunoreactive bands normalized to β-actin, and expressed as fold changes over the sham-operated controls. *p < 0.05 versus the I/R group. n = 6 per group.

Proinflammatory cytokines, like TNF-α and MCP-1, are known to stimulate the expression of MMPs that can digest tight junction and basement membrane proteins, contributing to the disruption of BBB (Dimitrijevic et al., 2006; Yang and Rosenberg, 2011; Yao and Tsirka, 2014). Elevated MMP-9 levels following stroke are associated with the disruption of BBB and brain edema (Rosenberg, 2012). To determine whether TMP improves the BBB permeability associated with downregulating the expression of MMP-9, the immunoblot assay was performed to examine the expression of MMP-9 in the ischemic brain in mice with or without the TMP treatment. As shown in Figure 4, the increased levels of MMP-9 were detected in the ischemic brain in both the TMP- and vehicle-treated groups compared to the sham-operated group. However, the elevated MMP-9 levels observed in the TMP group was significantly lower than that seen in the vehicle-treated group (Figure 4), suggesting TMP could effectively suppress the expression of inflammatory cytokines and MMP-9 in the ischemic murine brain.

### TMP Protects Against OGD-Induced Cell Damage is Abolished by Knockdown of MCPIP1

**FIGURE 5:**
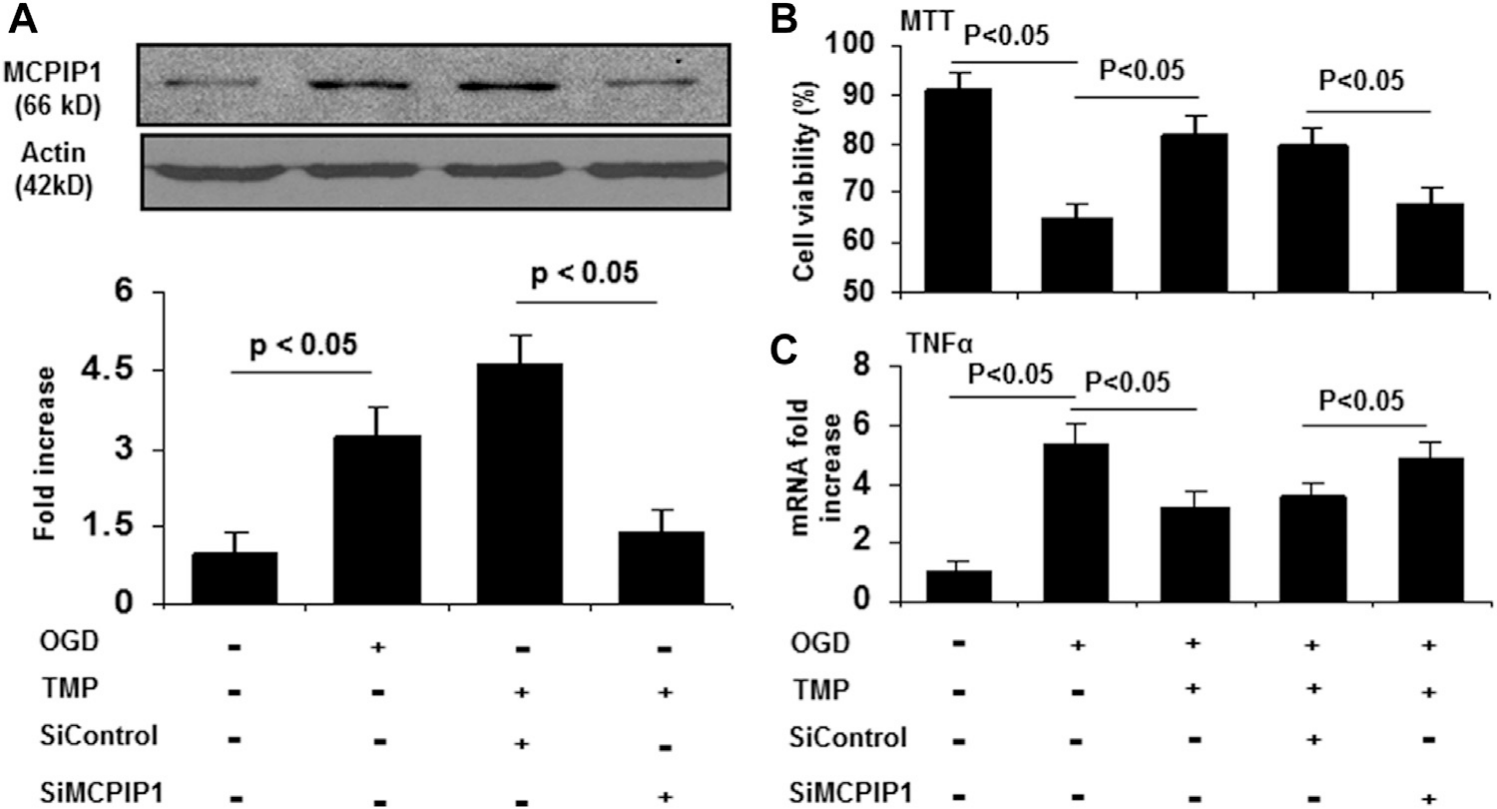
The neuroprotective effects of TMP is blocked by knockdown of MCPIP1 in vitro. The primary cultured mouse cortical neurons were pretreated with siRNA against MCPIP1 for 24 h, followed by treatment with TMP (10 μM) for 12 h. The cells were then subjected to oxygen-glucose deprivation for 24 h. Cell viability was assessed by MTT assays. mRNA levels for MCPIP1 and TNF-α were determined by qRT-PCR. Experiments were repeated three times. **(A)** Expression of MCPIP1 was detected by immunoblots **(*Upper panel*)**, and immunoreactive bands were quantified by densitometry analysis normalized to β-actin, and expressed as fold changes over the untreated controls **(*Lower panel*)** (n=3). **(B)** Cell viability was evaluated by MTT assays (n=3). **(C)** TNF-α mRNA levels were assayed by qRT-PCR (n=3).

Given that TMP reduces the infarct volume (Xiao et al., 2010; Kao et al., 2013) and upregulates MCPIP1 in the context of stroke in mice, it is reasonable to postulate that the neuroprotective effects of TMP may, at least in part, be related to the induction of MCPIP1. A siRNA experiment was performed in cultured primary neurons to determine the role of MCPIP1 in TMP-mediated protective effects *in vitro*. As shown in Figure 5A, siRNA MCPIP1 (siMCPIP1) significantly reduced the MCPIP1 expression induced by the TMP or the OGD, but a control siRNA (siControl) did not affect the MCPIP1 expression. The cultured neurons exposed to the OGD showed only 60.8% viability, and the TMP at a concentration of 10 µM protected the neurons against cell death induced by the OGD from 60.8 to 76.8% (*p* < 0.05). Cell viability offered by the TMP treatment, however, was significantly inhibited by knockdown of MCPIP1 with the MCPIP1-specific siRNA (Figure 5B). Moreover, the OGD-induced mRNA levels of TNF-α, a cytokine known to cause apoptosis, were inhibited by the TMP treatment, while this inhibition in TNF-α expression by TMP was abolished by MCPIP1-specific siRNA (Figure 5C).

## Discussion

TMP has been suggested to provide neuroprotection during ischemic stroke. Data presented here indicate that TMP significantly attenuated BBB permeability and brain edema accompanied by the upregulation of MCPIP1 and downregulation of pro-inflammatory cytokines, TNF-α, IL-1β, IL-6, MCP-1 as well as MMP-9 in the ischemic brain tissues *in vivo*. We further demonstrated that silencing of MCPIP1 using siRNA led to the enhancement of the expression of TNF-α and significantly blocked TMP-mediated cell protective effects *in vitro*. Our results suggest that the neuroprotective effects conferred by TMP are mediated, at least in part, *via* upregulation of MCPIP1 and downregulation of inflammatory responses in the ischemic brain.

Ischemic stroke is known to cause neuroinflammation that contributes to the progression of ischemic insult (Dimitrijevic et al., 2006; Strecker et al., 2011; Yao and Tsirka, 2014). An increase in levels of pro-inflammatory cytokines, such as TNF-α, IL-1β, IL-6, and MCP-1, are thought to trigger the early events that cause BBB breakdown and subsequent progression of neuron death and dysfunction (Dimitrijevic et al., 2006; Strecker et al., 2011; Yao and Tsirka, 2014; Amantea et al., 2015; Esenwa and Elkind, 2016). Upregulation of MMP-9 by pro-inflammatory cytokines has been implicated in the disruption of the BBB during hemorrhagic transformation and ischemic brain injury in stroke (Dimitrijevic et al., 2006; Yang and Rosenberg, 2011; Yao and Tsirka, 2014). TMP has been shown to effectively improve BBB permeability by the enhancement of peripheral cholinergic anti-inflammatory effects in traumatic brain injury (Wang et al., 2017). Also, TMP was reported to improve BBB permeability and neuronal damage in focal cerebral ischemia/reperfusion injury in rats (Tan et al., 2015). Consistent with the previous studies, we showed that administration of TMP before and after MCAO significantly improved the integrity of the BBB. As proinflammatory cytokines are implicated to cause disruption of the BBB, we observed that mRNA levels of TNF-α, IL-1β, IL-6, and MCP-1 were significantly elevated in the ischemic brain tissues, and these cytokines were significantly downregulated by the TMP treatment. Also, we demonstrated that the TMP inhibited the upregulation of MMP-9 in the ischemic brain. These results suggest that the improvement of the BBB permeability by the TMP may be closely related to the inhibition of the neuroinflammation occurred in the ischemic brain, which is in agreement with the previous report (Tan et al., 2015) and also supported by our findings that the TMP protects cultured neurons from OGD-induced death associated with the downregulation of TNF-α transcript *in vitro*.

MCPIP1 is a newly identified zinc-finger protein that was reported as an endogenous modulator of inflammation. (Liang et al., 2008; Matsushita et al., 2009; Liang et al., 2010) Both *in vitro* and *in vivo* studies have shown that the induction of MCPIP1 is an important cellular protective mechanism against inflammatory injury. (Liang et al., 2008; Matsushita et al., 2009; Liang et al., 2010) MCPIP1 is now considered as a negative regulator of inflammatory responses, not only by inhibition of NF-κB activation but also inhibition of inflammatory signal pathways by regulating mRNA degradation, miR synthesis and IL-17 receptor degradation as well. (Liang et al., 2008; Matsushita et al., 2009; Liang et al., 2010) Moreover, MCPIP1 expression is significantly increased in ischemic lesions in the murine myocardium (Niu et al., 2015) and brain (Jin et al., 2013). Overexpression of MCPIP1 attenuates post-infarct cardiac remodeling and preserves cardiac function in a mouse model of myocardial infarction (Labedz-Maslowska et al., 2015; Niu et al., 2015). MCPIP1 deletion causes increased infarct volume and inflammatory gene expression in transient middle cerebral artery occlusion in mice (Jin et al., 2013). Here, we found that the TMP upregulates MCPIP1 associated with reduced inflammatory gene expression and preserved the integrity of BBB. The expression of MCPIP1 protein by the TMP was not parallel by its mRNA expression. The discrepancy between the mRNA and protein levels of MCPIP1 may be attributed to translational efficiency or posttranslational regulation. This hypothesis is supported further by a recent report discussing a transcriptional regulation of MCPIP1 (Uehata and Akira, 2013). In addition, we showed that genetic suppression of MCPIP1 abolishes TMP-mediated cell protection, upregulates TNF-α expression, and exacerbates OGD-induced cell death in cultured neurons *in vitro*. The results obtained from our study are consistent with a recent study showing that genetic knockout of MCPIP1 exacerbates ischemia-induced BBB breakdown and ischemic brain injury (Jin et al., 2013; Jin et al., 2019b). Other proinflammatory cytokines, like IL-1β, IL-6, and MCP-1, also contribute to the BBB breakdown and subsequent progression of neuron death and dysfunction (Dimitrijevic et al., 2006; Strecker et al., 2011; Yao and Tsirka, 2014; Amantea et al., 2015; Esenwa and Elkind, 2016). A lack of data for levels of IL-1β, IL-6, and MCP-1 by knockdown of MCPIP1 is a limitation of this study.

In conclusion, our data show that TMP upregulates MCPIP1 in the murine brain and preserves the BBB integrity in mice. The mechanisms underlying this protection could be associated with the downregulation of MMP-9 and inflammatory cytokines in the murine brain. Our findings demonstrate the anti-inflammatory properties of TMP and suggest that the underlying mechanism may be mediated, at least in part, *via* upregulation of MCPIP1. Given its anti-oxidative, anti-apoptosis, and anti-inflammatory effects (Ran et al., 2011; Sun et al., 2012; Kao et al., 2013; Yang et al., 2017), TMP is one of the most commonly used drugs in clinical practice in China, and has been used in the treatment of cardiovascular and cerebrovascular diseases, such as coronary heart disease, cerebral thrombosis, and vasculitis (Guo et al., 1983). In recent years, TMP has been reported for the treatment of kidney disease, portal hypertension, type 2 diabetes, and tumors (Zhao et al., 2016). TMP is currently on clinical trial to determine its efficacy and safety in the treatment of pulmonary hypertension (Chinese Clinical Trial Register, ChiCTR1800018664) (Chen et al., 2019). Our finding that TMP induces MCPIP expression reveals a potential therapeutic target for TMP, and therefore warrants the need for future studies on the molecular mode of action of TMP relevant to clinical application.

## Data Availability

The raw data supporting the conclusions of this article will be made available by the authors, without undue reservation.
